# The Role of Genome Accessibility in Transcription Factor Binding in Bacteria

**DOI:** 10.1371/journal.pcbi.1004891

**Published:** 2016-04-22

**Authors:** Antonio L. C. Gomes, Harris H. Wang

**Affiliations:** 1 Department of Systems Biology, Columbia University, New York, New York, United States of America; 2 Department of Pathology and Cell Biology, Columbia University, New York, New York, United States of America; National Center for Biotechnology Information (NCBI), UNITED STATES

## Abstract

ChIP-seq enables genome-scale identification of regulatory regions that govern gene expression. However, the biological insights generated from ChIP-seq analysis have been limited to predictions of binding sites and cooperative interactions. Furthermore, ChIP-seq data often poorly correlate with *in vitro* measurements or predicted motifs, highlighting that binding affinity alone is insufficient to explain transcription factor (TF)-binding *in vivo*. One possibility is that binding sites are not equally accessible across the genome. A more comprehensive biophysical representation of TF-binding is required to improve our ability to understand, predict, and alter gene expression. Here, we show that genome accessibility is a key parameter that impacts TF-binding in bacteria. We developed a thermodynamic model that parameterizes ChIP-seq coverage in terms of genome accessibility and binding affinity. The role of genome accessibility is validated using a large-scale ChIP-seq dataset of the *M*. *tuberculosis* regulatory network. We find that accounting for genome accessibility led to a model that explains 63% of the ChIP-seq profile variance, while a model based in motif score alone explains only 35% of the variance. Moreover, our framework enables *de novo* ChIP-seq peak prediction and is useful for inferring TF-binding peaks in new experimental conditions by reducing the need for additional experiments. We observe that the genome is more accessible in intergenic regions, and that increased accessibility is positively correlated with gene expression and anti-correlated with distance to the origin of replication. Our biophysically motivated model provides a more comprehensive description of TF-binding *in vivo* from first principles towards a better representation of gene regulation *in silico*, with promising applications in systems biology.

## Author Summary

A quantitative description of transcription factor (TF) binding *in vivo* is critical for our understanding of gene regulation. Chromatin Immunoprecipitation followed by sequencing (ChIP-seq) provides a genome-scale map of TF-binding. However, a quantitative characterization of the impact of genome accessibility on TF-binding in bacteria remains elusive. In order to help recruit or block gene expression, TFs must have physical access to regulatory regions. This paper presents a thermodynamics model that describes TF-binding in terms of genome accessibility and binding site affinity. We apply this model in a ChIP-seq dataset for *Mycobacterium tuberculosis* and observed that genome accessibility is critical to our understanding of TF-binding *in vivo*. This new model provides practical applications, such as *de novo* prediction of TF-binding peaks and a framework to measure DNA accessibility from ChIP-seq data. Our model enables us to quantify the relationship of genome accessibility with genomic features and suggest mechanisms that influence genome accessibility *in vivo* (e.g. distance to oriC). The model proposed in this study gives new perspective for ChIP-seq analysis in bacteria towards an improved description of gene regulation *in silico*.

## Introduction

In order to adapt to different environmental challenges, microorganisms need to precisely control the expression of specific sets of genes at defined magnitudes at any given moment [1, 2]. This control is mediated by regulatory proteins such as transcription factors (TF) that are able to recognize and bind specific DNA sequences to recruit or block the gene expression machinery. Recent advances in next-generation sequencing have now enabled us to measure TF-binding *in vivo* at the genome scale [3–5].

Chromatin Immunoprecipitation followed by sequencing (ChIP-seq) is a popular technology for *in vivo* measurements of TF binding [6–8], which uses TF-specific antibody selection and high-throughput sequencing to identify the genomic regions that are bound by a query TF. In parallel, technologies for high-throughput characterization of TF-binding *in vitro* have also emerged [9–13]. Yet, only a fraction of the expected binding sites are bound under physiological conditions [8] and *in vivo* measurements are poorly correlated with *in vitro* ones [14, 15].

TF-binding *in vivo* is often more complex than what can be measured *in vitro* due to multiple factors [16]. For instance, strength of TF-binding affinity [17, 18], presence of multiple binding sites [19], cooperative interactions [18, 20], and genome accessibility [21, 22] have all been shown to impact TF-binding *in vivo*. Incorporating these parameters in ChIP-seq analysis can lead to more accurate models of gene regulation across the whole genome [14, 15].

As sequencing costs continue to decrease, challenges in ChIP-seq studies are transitioning from data generation to analysis and modeling [23]. Data analysis methods have moved from purely peak identification to physically-motivated models of ChIP-seq coverage [24]. Early computational methods focused on identifying statistically enriched peaks that correspond to TF-binding regions [5, 25–28]. Recent methods are incorporating mechanistic principles to extract regulatory insights [24, 29–31]. For example, the BRACIL method integrates ChIP-seq coverage, motif score, and thermodynamic modeling through a signal processing representation to predict binding site locations with high-resolution as well as cooperative interactions [24]. The growing abundance of ChIP-seq data creates a greater demand for more comprehensive models [15, 23, 32] and an opportunity to evaluate key parameters of TF-binding *in vivo*.

Within the cell, transcription factors need to have physical access to the relevant regulatory regions in order to control gene expression. In eukaryotes, genome accessibility is mostly caused by different chromatin states due to epigenetic factors such as histone modification and nucleosome structures [33]. The chromatin state can lead to gene silencing throughout the genome and have been used to estimate genome accessibility. In contrast, bacteria do not organize their genome in nucleosomes, thus genome accessibility is a subtle feature that is hard to be measured. In general, accessibility is not uniform across the genome due to the presence of global factors such as nucleoid associated proteins (NAPs) that alter genomic architecture [15, 21, 22] or local factors such as presence of repressor elements that block recruitment of RNA polymerase [21, 34]. Alteration of global genome structure can lead to changes in gene expression [35, 36]. For example, NAPs are associated with highly expressed genes that are organized into transcription factories [21]. The challenges in measuring and estimating genome accessibility have impeded the incorporation of this feature into bacterial ChIP-seq analysis.

Here, we present a novel biophysically motivated model that incorporate genome accessibility and highlights its importance in assessing TF-binding in bacteria. Extending our previous efforts to mechanistically characterize ChIP-seq coverage information [24], our model treats ChIP-seq binding profiles as a Boltzmann distribution with two parameters: genome accessibility and binding affinity. We applied this model on a large-scale dataset used to map the regulatory network of *M*. *tuberculosis* and compared the results to a simplified model that only considers binding affinity. Our results show that genome accessibility can explain variability in ChIP-seq coverage and peaks, and is associated with specific groups of gene function.

## Results

### Biophysically motivated model of TF-binding *in vivo*

Using ChIP-seq data, biophysically motivated models can provide a quantitative framework for determining key parameters of *in vivo* TF-binding. We represent the ChIPseq profile in region bins of 500 bp and look for the influence of genome accessibility in TF binding. From a thermodynamic perspective, the probability, *p*_*ij*_, that a TF *j* binds to a genome region *i* depends on the affinity between the TF and the specific sequence it binds, *w*_*ij*_, as well as on the degree that this region is accessible, *a*_*i*_. Formally, the probability of binding is defined by the following equation (see Methods for detailed derivation):

$$log\left(p_{ij}\right)=a_{i}+w_{ij}$$ (1)

**Fig 1:**
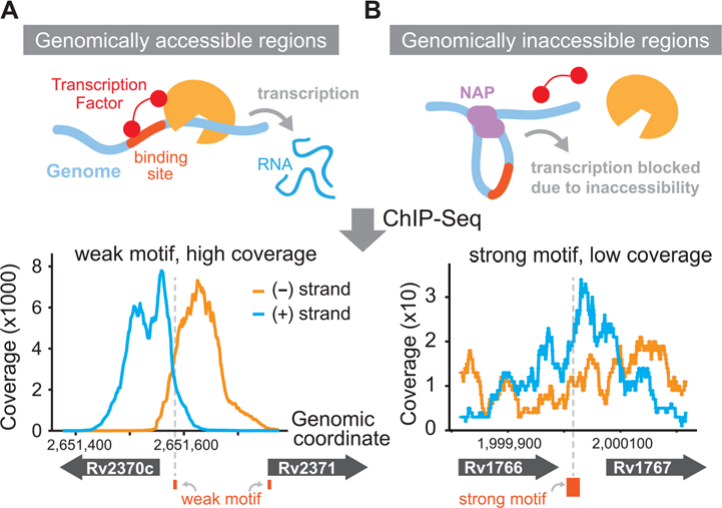
The role of genome accessibility in TF-binding *in vivo*. The genome accessibility model differentiates genomic regions as accessible **(A)** or not accessible **(B)**. ChIP-seq data show that coverage cannot be explained by binding affinity alone. Example data is shown for an accessible region **(A)** that has a weak binding site (small purple box, p-value ~ 5x10^-4^) and high ChIP-seq coverage. The gray dashed line indicates the location of the TF-binding site motif. Example data is shown for an inaccessible region **(B)** with a strong binding site (big purple box, p-value ~ 5x10^-6^) but low coverage. Example data shown are for *M*. *tuberculosis* DosR ChIP-seq experiments [15].

TF-binding is represented in terms of binding affinity alone by constraining *a*_*i*_ *= 0* for all *i*. The accessibility parameter is inferred indirectly by performing linear regression on a large-scale dataset of ChIP-seq experiments [15, 32]. The affinity parameter is obtained from the motif score. The parameter *a*_*i*_ describes a global trend in the probability of binding to region *i* by any TF. Here, we refer this as the genome accessibility for better biological interpretation of the results. Fig 1A and 1B illustrates schematically how genome accessibility influences TF-binding. Eq 1 is motivated by the poor correlation between ChIP-seq coverage and motif score (S1 Fig). For example, genomic regions with weak motif scores are observed with strong binding signal and vice versa (Fig 1).

### Genome accessibility improves ChIP-seq interpretations

**Fig 2:**
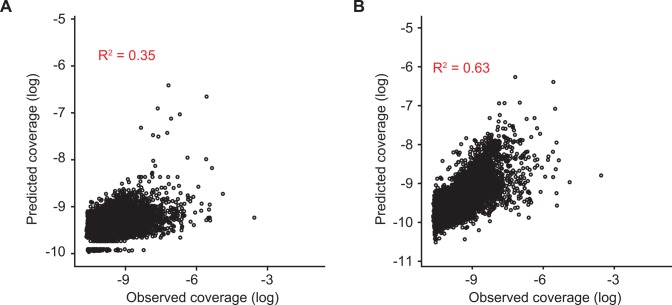
Genome accessibility improves prediction of ChIP-seq profiles in comparison to a model that only considers motif score. Motif score alone explains only 35% of the observed variance **(A)**, while the improved biophysically motivated model that incorporates genome accessibility explains 63% of the variance **(B)** (p<10^−16^, likelihood ratio test). The predicted coverage is estimated from parameters fitted for Eq 1. Coverage is represented in terms of log(*p*_*ij*_). The panels display a subset of 10000 points that was randomly selected to reduce the density of points and improve visualization.

We evaluated the extent to which genome accessibility can explain ChIP-seq data. We model our data according to Eq 1 and use a linear fixed effect model to estimate parameters and predict ChIP-seq profiles. The dataset comprises ChIPseq data for a total of 64 unique TFs obtained under same protocol and growth condition (see Methods). The ChIP-seq profile for a specific TF is defined as the normalized abundance of sequence reads that align to each region. The result suggests that the accessibility parameter is a global trend that provides preferential binding on specific genomic regions. We observe that genome accessibility improves prediction of ChIP-seq profiles when compared to a model that considers only binding site affinity. Quantitatively, the accessibility model explains 63% of the observed variance, while motif score alone explains only 35% (p-value <10^−16^, Fig 2). We also explored a more complex representation for binding affinity that considers best motif match, number of binding motifs and a combined score for all motif matches. The combined score is defined as the sum of *-log(pvalue)* for all motif matches. The accessibility values estimated by the more complex model is almost the same as the one estimated by the model that considers only best motif match (correlation above 99.9%; S8 Fig).

### Prediction of ChIP-seq peaks *de novo*

Our model can predict functional features that are useful in ChIP-seq analysis. The most common task in ChIP-seq analysis is the identification of TF-binding peaks, i.e. genomic regions that are bound by the TF under query, which shows a peak in ChIP-seq coverage [5, 28]. We classify regions into two groups: peaks or not peaks, according to peak-caller method described in previous work [15]. Each region is ranked with a score that indicates how likely they are to contain a peak. Given a threshold, false positives represent regions classified as peak by peak-calling but labeled as not peaks by the ranking score for *de novo* peak prediction. Similarly, false negatives represent regions that are classified as not peaks by peak-calling but labeled as peaks by the ranking score for *de novo* peak prediction. The rank for peak classification is defined according to motif and accessibility score and used to construct the ROC curve. Motif score is defined as the maximum *log(p-value)* of motif match per region bin and accessibility score is the estimated value for parameter *a*_*i*_ from Eq 4. We consider three models for peak classification: *motif only*, *motif plus accessibility*, and *normalized motif plus normalized accessibility*. The first model predicts peaks using only motif score obtained by motif scan; the second model uses the sum of motif score and accessibility value; the last model rescale the values of motif score as well as accessibility in the interval from 0 to 1 and use their sum for peak prediction (see Methods).

**Fig 3:**
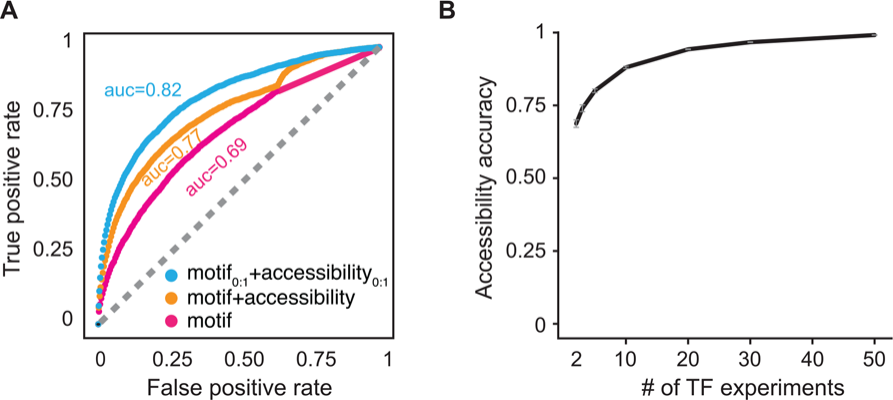
Genome accessibility improves binding peak prediction in ChIP-seq profiles. Reference ChIP-seq peaks are defined according to method previously described [15]. A receiver and operator characteristic curve is shown in panel **(A)**. Three models are presented for *de novo* peak prediction (see main text for details). The accessibility parameter (blue and orange lines) increases peak prediction from 0.69 to 0.82 in comparison to a model that only accounts for motif score (violet-red line). **(B)** Accuracy of genome accessibility estimation as a function of number of ChIP-seq experiments. The accuracy of accessibility values is defined as the Pearson correlation between the estimated values for a subset of ChIP-seq experiments and the one estimated for entire dataset (S2 Fig). The expected accuracy of accessibility values is defined as the mean value of 100 samples. Error bars represent one standard error.

The results show that DNA accessibility improves *de novo* ChIP-seq peak predictions when compared to predictions that consider motif only. As measured by the area under a receiver operating characteristic (ROC) curve, *de novo* ChIP-seq peak prediction occurs with values 0.69, 0.75, and 0.82 for method that uses motif only, motif score plus accessibility, and normalized motif score plus normalized accessibility, respectively (Fig 3A). The affinity values are sequence specific and by definition do not dependent on experimental conditions while the accessibility parameters may vary depending on experimental condition (S9 Fig). Therefore, given that TF-binding affinity score is previously known, one would only need to measure genome accessibility to predict TF-binding under novel growth conditions or for TFs with known binding motifs. This rationale can significantly reduce the need for additional ChIP-seq experiments.

The ability to predict ChIP-seq peaks *de novo* depends on the robustness of the genome accessibility metric and the ease to estimate its parameters under novel experimental conditions. The robustness of DNA accessibility values is illustrated by plotting the accuracy of accessibility values as a function of dataset size used for their estimation, i.e. the expected Pearson correlation between the accessibility estimated in a subset of given size versus the accessibility estimated in the entire dataset (S2 Fig). The expected accuracy for the accessibility values is estimated from 100 distinct samples for each subset size. We observe that as low as 10 ChIP-seq experiments is sufficient to estimate the accessibility values with ~90% accuracy (Fig 3B and S2 Fig).

The global trend in genome accessibility is robust to overexpression of a single TF. The ChIP-seq experiments used in this analysis were obtained under the same experimental set, with the exception that the TF under query was overexpressed [15]. We observe that removing any single TF from our dataset does not affect the estimated accessibility value (correlation between estimates are >99%). This indicates that the estimation of genome accessibility is robust to single TF overexpression. Moreover, we observe that just a few ChIP-seq experiments are sufficient to estimate genome accessibility with high correlation to its reference value. Only two ChIP-seq experiments are sufficient to estimate accessibility values with expected 0.7 correlation to the reference (Fig 3B and S2 Fig). We also observed that binding profile of some TFs are better correlated with the estimated accessibility values (S4 Fig). This result may indicate TFs that play a key role on genome structure or good candidates to infer genome accessibility.

### Genomic features related to genome accessibility

**Fig 4:**
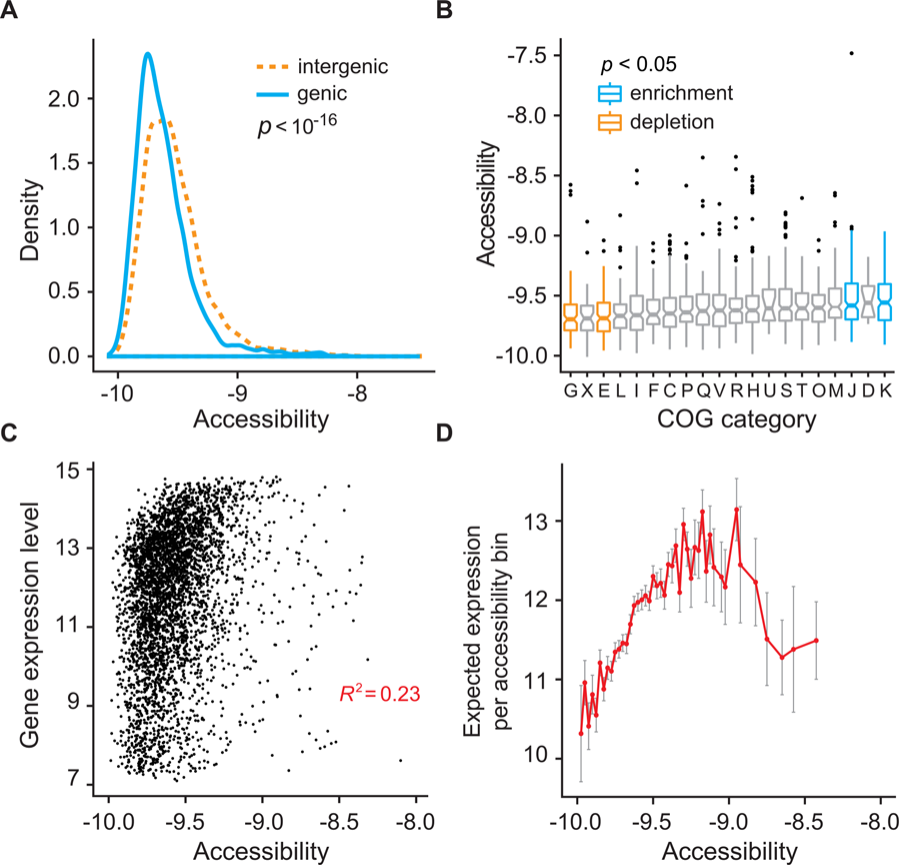
Genome accessibility correlates with genomic features. **(A)** Intergenic regions are more accessible than protein coding genic regions (p<10^−16^). **(B)** Regions associated with amino acid and carbohydrate metabolism and transport (COGs E and G) show statistically reduced accessibility. Genes associated with transcription and translation (COGs K and J) show statistically higher accessibility (p<0.05, Bonferroni correction). **(C)** Gene expression is positively correlated with accessibility. The correlation of DNA accessibility with gene expression after controlling for values of motif affinity is 0.278 (p<3.98 10^−56-^; function pcor and pcor.test, R package *ggm*). **(D)** Expected gene expression is highest at an intermediate level of accessibility. Accessibility bins with less than 10 data points are clustered with the neighboring bin with fewer data points. Error bars represent one standard error from the mean.

Our model can be used to measure the accessibility state of each region in the genome. We sought to determine if genome accessibility is associated with various genomic features. Consistent with previous studies [37], intergenic regions are more accessible than protein coding regions (Fig 4A). Genome accessibility also appears to vary between genes or their regulatory regions based on their Clusters of Orthologous Groups (COG) assignments. In particular, genes or their regulatory regions in COGs for metabolism and transport of amino acids (COG category E) as well as carbohydrates (COG category G) are less accessible, while COGs for translation (COG category J) and transcription (COG category K) are more accessible (p<0.05 after Bonferroni correction; see Fig 4B). The observation of higher genome accessibility in transcription and translation genes is consistent with previous observations that DNA structure plays a critical role in expressing rRNA operons [21, 38, 39]. Finally, we observe that expression levels are positively correlated with genome accessibility (*R*^2^ = 0.23, Pearson correlation, Fig 4C). Interestingly, our results show that the expected expression level is the highest at intermediate values of genome accessibility (Fig 4D), which suggest that there may be a non-linear relationship between accessibility and gene expression.

**Fig 5:**
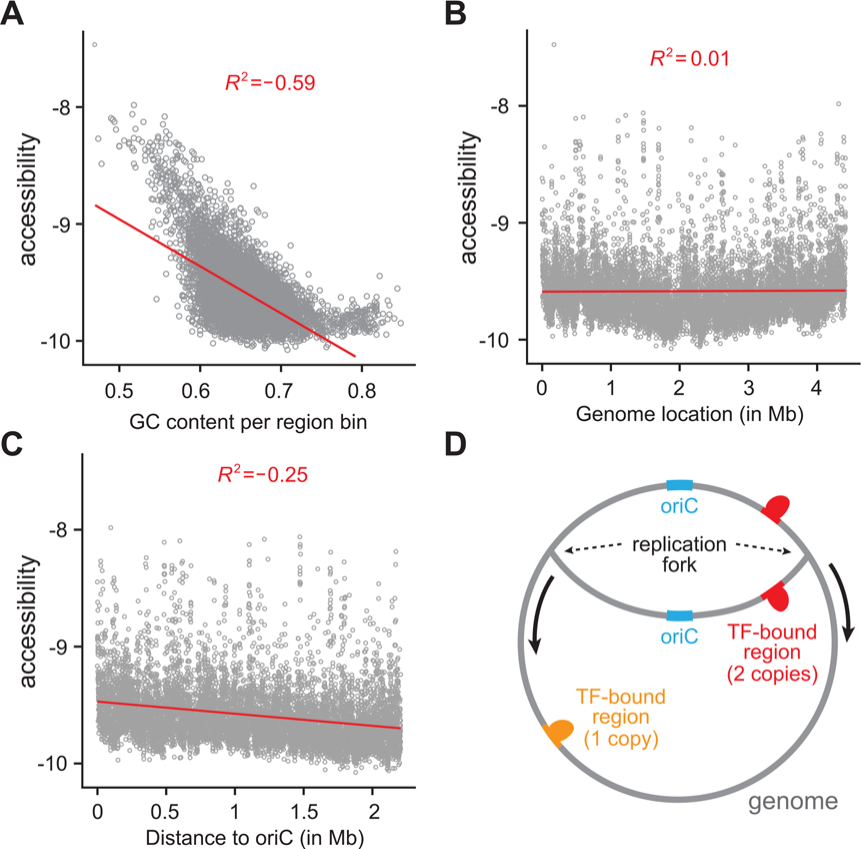
Genome accessibility is affected by GC content and distance to oriC. **(A)** Accessibility is negatively correlated with local genomic GC content. The correlation between accessibility values and region GC content after controlling for values of motif affinity is -0.30 (p < 10^−179^; function pcor and pcor.test, R package *ggm*). **(B)** Accessibility does not appear to correlate with genome position. **(C)** Accessibility is negatively correlated with distance to oriC. **(D)** A schematic of genome replication that could explain the correlation between accessibility and distance to origin of replication.

Furthermore, our analysis shows that genome accessibility is biased by genomic position and GC content (Fig 5). Accessibility has a strong negative correlation with GC content (Fig 5A). In addition, accessibility is negatively correlated with distance to the origin of replication, oriC (Fig 5C), while no apparent correlation is observed in comparison to genome position alone (Fig 5B). This suggests two possible mechanisms that may influence genome accessibility: (i) DNA replication makes genomic regions more accessible for TF-binding, or (ii) there is a higher copy number of genomic regions near the oriC, leading to an apparent increase in genome accessibility (Fig 5D). These two mechanisms are not necessarily mutually exclusive and would be interesting to explore in future studies.

## Discussion

In this study, we developed a biophysically motivated formulation for bacterial ChIP-seq analysis that contributes to new biological insights of the role that genome accessibility plays in bacterial gene regulation. The model highlights the importance of binding affinity and genome accessibility for *in vivo* TF-binding. The model formulates the TF-binding process in thermodynamic terms and derives a linear relationship between accessibility, binding affinity, and probability of binding. This relationship enables us to estimate the model parameters from ChIP-seq data. We optimized our statistical framework with a fixed-effects representation to make parameter estimation more computationally efficient.

Numerous studies have investigated the role of genome accessibility on TF-biding in eukaryotic organisms [30, 40–44]. However, to the best of our knowledge, the work described here is the first attempt for a genome-scale quantitative measurement of DNA accessibility in bacteria. In eukaryotes, reads from DNAse I assays are well-correlated with binding regions [40, 41]. Pique-Regi et al. reported that DNAse I assays can inform genome accessibility for predicting ChIP-seq peaks from ENCODE data using a Bayesian probabilistic model that integrates accessibility with motif information from position weight matrix (PWM), TSS location and evolutionary conservation [29]. Other studies [43, 44] used a threshold on the coverage of DNAse I signal was used to distinguish accessible from silent genome regions and infer TF-TF interaction as well as set of TFs that drive tissue, cell type, and developmentally specific gene expression patterns in *Drosophila*. Foat et al. developed a thermodynamics model of binding based on equilibrium dissociation constant between bound and unbound states and used a least square regression model to infer binding affinity from ChIP-chip data of *Saccharomyces cerevisiae [42]*. However, genome accessibility was not considered in the model. Peng et al. developed another thermodynamic model that includes accessibility and binding energy to predict expression dynamics in *Drosophila* [30]. Accessibility was inferred from DNAse I assays and model parameters were trained based on an objective function that rewards good fit on highly expressed bins.

The method proposed in this paper has several novel features in comparison to those outlined above for eukaryotes. In contrast to eukaryotic genome accessibility models, which are inferred directly from DNAse I assays, our method infer accessibility from binding profiles of multiple ChIP-seq characterized TFs. Our thermodynamics model of TF-binding is derived in terms of binding affinity and genome accessibility by using Lagrange multipliers and free energy of Helmholtz (see Methods). A mixed effects linear regression model is used to make fit efficient and computationally feasible. In addition, the quantitative assessment of DNA accessibility in bacteria provides the possibility of testing hypothesis, novel biological insights, and applications.

The framework described here could be used to assess TF-binding using a reduced set of necessary ChIP-seq measurements. Instead of collecting ChIP-seq data for each TF in every new experimental condition, one would only need to perform a small set of experiments to estimate the state of genome accessibility. Then, in combination with established TF affinity data, one can accurately predict TF-binding genome-wide as demonstrated here. This approach could link both *in vitro* and *in vivo* experimental datasets under a unifying framework. Our model provides a step forward in our ability to infer TF-binding at different growth states *in silico* to capture the dynamic nature of gene regulation in bacteria.

Biophysical processes *in vivo* as well as experimental protocols should be considered for proper interpretation of accessibility values. Variance in DNA structure, binding competition, or *in vitro* artifacts in immuno-precipitation affects the measured genome accessibility. NAPs can shape genome structure at a global scale, while specific genome modification factors can affect accessibility within a particular regulon. Multiple transcription factors that bind to the same genomic region may lead to binding competition, causing a decrease in the observed accessibility. Variations in immuno-precipitation protocols and inherent noise in the technique may lead to variation in the estimation of binding specificity and sensitivity. These and other factors may cause genome accessibility to contain bias from ChIPseq experiments and could be helpful in providing better background estimation.

Ultimately, the importance of accessibility in bacteria genome remains to be further explored. In eukaryotic cells, genomic accessibility is critical in fine-tuned gene regulation [45] through controlled activation [46], minimizing biological noise [47], and providing epigenetic regulation [33]. These processes may be similarly important in bacteria physiology. For instance, genomic accessibility could cause stochastic gene expression and influence cell fate [48]. Engineering or altering genome accessibility may lead to new approaches in synthetic gene regulation and advance research in systems and synthetic biology [33].

Our work highlights that new biological insights can be obtained through biophysically-motivated mechanistic models of gene regulation. This approach should inspire more refined models of cellular physiology and adaptation. Here, we showed that thermodynamic principles can improve our understanding of TF-binding and genomic structural states. More refined models that integrate accessibility and binding affinity with other factors such as cooperative interaction and multiplicity will enhance our understanding of gene regulation, which will lead to a more comprehensive representation of whole cell physiology [49].

## Methods

### Thermodynamics model of gene regulation

The probability of TF-binding to a specific region is represented as a Boltzmann distribution that depends on two parameters: accessibility and affinity. The accessibility parameter, *a*_*i*_, is specific to the DNA region and represents how likely a region *i* is to be bound by any transcription factor. The affinity parameter, *w*_*ij*_, represents the specific affinity between a transcription factor *j* and a region *i*. Formally, the probability that a TF *j* binds at region *i*, *p*_*ij*_, is defined as:

$$p_{ij}=e^{a_{i}+w_{ij}}$$ (2)

This representation omits negative signs and the temperature parameter because they are not relevant to the approach in this study. In thermodynamic terms, Eq 2 represents a grand-canonical ensemble in which each region bin can exchange particles (i.e. TFs) and energy. The parameter *a*_*i*_ represents the chemical potential in region *i* and the parameter *w*_*ij*_ represents the energy associated with TF binding (see S1 Text for detailed mathematical derivation).

The probability *p*_*ij*_ can be measured directly from the ChIP-seq data. In order to make this parameter robust and independent on the sequencing depth, we define *p*_*ij*_ as

$$p_{ij}=\frac{C_{i,j}}{\sum_{i}C_{i,j}}$$ (3)

where the coverage parameter, *C*_*i*,*j*_, represents the number of reads from experiment *j* that lies in region *i*. A formal definition for region bins is presented in the next section.

### Linear regression representation

Eq (2) can be transformed to a linear representation. This representation is shown in Eq 1 in the main text and repeated here for clarity:

$$\text{log}\left(p_{ij}\right)=a_{i}+w_{ij}$$ (4)

Eq 4 permits that we use simple linear regression to estimate the parameters that determine ChIP-seq profiles.

This study is restricted to TFs whose binding sites can be summarized by a position weight matrix (PWM). Motif PWM was obtaining as the output of BRACIL [24]. The PWM provides a first order approximation of the affinity between the TF and the region it binds [10, 50]. We call *s*_*i*,*j*_ the affinity score of TF *j* to region *i* estimated according to the PWM. This approximation can be placed in Eq 4, and simplify linear regression as following:

$$\text{log}\left(p_{ij}\right)=a_{i}+t_{j}\cdot s_{ij}$$ (5)

The parameter *t*_*j*_ is a constant that represents underlying variables specific to each ChIP-seq experiment, such as TF concentration, ChIP-seq coverage as well as quality of immuno-precipitation. The affinity score, *s*_*i*,*j*_, is defined as the -log_10_(p-value) of motif match with highest score in region *i*. Motif scan is performed using FIMO [51]. A affinity score of 2 was given to regions without any motif match.

We assume binding affinity to the sequence decreases monotonically with motif *p-value*. The p-value indicates the probability a score as good as the one observed in motif match occurs by chance according to the reference motif PWM. Thus, the binding affinity is monotonically correlated with–log10(p-value) of a motif match. By expanding it in Taylor series, the term–log10(p-value) becomes a first order approximation for binding affinity that suffices for the purpose of this research.

The genome is binned in regions of 500 bp to create a standardize profile and enable comparison of multiple TF experiments simultaneously. Cases with very low coverage are removed from analysis. In numbers, we classified the *M*. *tuberculosis* genome in 8824 region bins and only considered data points in which log(p_ij_) > -10. Our rationale is to set up a threshold that considers data points that are informative for analysis and remove noisy ones that decrease the quality of genome accessibility estimation. 82.5% of the data points are used for analysis after applying the threshold of *log(p*_*i*_*) >-10*. This choice is supported by a sensitivity analysis that considers a wide range of minimum coverage threshold (S5–S7 Figs). The results are also robust for varying size of region bins (S10 Fig).

### Linear regression optimization

We optimized the statistical representation of Eq 5 to make the analysis practical and more efficient. The naïve approach would be to solve Eq 5 by a simple least square minimization. However, the number of data points and parameters needed would exceed 10^6^ and 10^4^, respectively. The least square minimization by QR decomposition (function *lm* in R) is impractical and we used a linear mixed-effects model (function *lmer*, R package *lfe*) instead.

The linear mixed-effects model optimizes regression because the parameter related to regional accessibility can be described as a random effect that shift the intercept of the probability of binding. As most parameters of Eq 5 correspond to the accessibility value of a region bin, the linear mixed-effects representation makes computation much more efficient.

In *lmer* annotation, our model uses the following formula: `*log(p) ~ s·t + (1|region_bin)*`, where *p*, *s*, and *t* are general representation of the corresponding parameters in Eq 5 and `*(1|region_bin)`*represents the random effect caused by accessibility to each region bin. The model that considers binding affinity only is represented as: `*log(p) ~ s·t*`.

### *De novo* peak prediction

We use three methods for *de novo* peak prediction: motif only, motif + accessibility and normalized motif + normalized accessibility. *Motif only* rank regions according to best motif match. *Motif + accessibility* sums the score of motif match (in terms of -log10(pvalue)) with the accessibility values estimated from fitting Eq 1 in the data. Finally, we define the minimum score to be 0 and maximum score to be 1 and re-scale motif as well as affinity score accordingly. This sum of the re-scaled score is used to rank regions for the method *normalized motif + normalized accessibility*.

### ChIP-seq data

The ChIP-seq data used for this analysis was obtained from a large-scale study that mapped the regulatory network of *M*. *tuberculosis* [15, 32]. The TF under query was FLAG-tagged and over-expressed under control of a mycobacterial tetracycline-inducible promoter. The enriched regions were computed according to the log-normal background model described in [15]. The binding motif was obtained as the output of the algorithm BRACIL [24], which uses MEME [51] to perform motif identification. FIMO [51] was then used to scan for binding sites at each region. Only TFs that recognize a binding motif with E-value < 10^−5^ were selected for this analysis. This resulted in a total of 99 ChIP-seq experiments that comprises 64 TFs.

Gene expression was defined as the median expression from the set of TF overexpression data, as described previously [15, 52].

COG categories were obtained from ftp://ftp.ncbi.nih.gov/pub/COG/COG2014/data and mapped to H37rv loci according to GENBANK annotation.

### Code availability

The code and corresponding documentation are available at https://sourceforge.net/projects/brasolia.

## Supporting Information

S1 Fig ChIP-seq profile is poorly correlated with binding affinity. **(A)** Motif for the transcription factor DosR (experiment label: Rv3133c_B121) is shown. This motif was predicted by BRACIL and used to estimate binding site affinity (see Methods). Correlation between binding site affinity and ChIP-seq coverage is shown in linear scale **(B)** and log scale **(C)**. The Pearson correlation is 0.19 and 0.34, respectively. Coverage and motif is computed per region bin. Coverage represents normalized sum of reads at each region bin and motif represents match with best score.
(EPS)

S2 Fig Correlation between reference accessibility with accessibility estimated in subsets of ChIPseq data of different sizes. The reference accessibility values are estimated by using the entire dataset. We show instances of accessibility values estimated from a subset of size 2 (A), 5 (B), 10 (C), 20 (D), 30(E), and 50 (F) TFs.
(EPS)

S3 Fig Variance of accessibility values per genome location. Each point shows the variance of accessibility for groups of 100 region bins.
(EPS)

S4 Fig Correlation between accessibility values and ChIP-seq coverage. We show the correlation between accessibility values and the ChIP-seq coverage per experiment.
(EPS)

S5 Fig The estimated accessibility values are robust to varying threshold of minimum coverage per region. We assessed the accessibility values by defining a minimum coverage per region threshold of 1000 (**A**), 5000, (**B**), 10000 (**C**), 20000 (**D**), and 35000 (**E)**. The fraction of data points included per threshold is 99.1%, 97.2%, 92.9%, 80.0% and 46.6%, respectively. Ideally, threshold should be strong enough to filter noisy data points, but not too stringent to filter informative points. The correlation with reference accessibility values (obtained from threshold *log(p*_*ij*_*) > -10)* is greater than 95% for cases with a moderate threshold (**B-D**). Regions with very low coverage reduce the quality of the estimated accessibility. This is observed when using a minimum threshold of 1000 (i.e. 2 units per bp) (**A**).
(EPS)

S6 Fig *De novo* peak prediction is robust to different values of minimum coverage per region threshold. The ROC plot for *de novo* peak prediction is shown for estimations obtained by using a minimum coverage threshold of 1000 (**A**), 5000 (**B**), 10000 (**C**), 20000 (**D**), and 35000 (**E)**. A very low minimum coverage per region threshold, e.g. 1000, reduces the predictive power for *de novo* peak prediction (**A**). The plot represents ROC curve from a subsample of 10000 data points. The box shows area under the curve (AUC) for each case. The standard error for all AUC values is less than 0.002.
(EPS)

S7 Fig Correlation of genome accessibility and distance to *OriC* is preserved for different values of minimum coverage per region threshold. The panels show results for estimations obtained by using a minimum coverage threshold of 1000 (**A**), 5000 (**B**), 10000 (**C**), 20000 (**D**), and 35000 (**E)**. The relationship between genome accessibility and distance to *OriC* is sharply reduced but still significant when using very inclusive threshold for minimum coverage per region equal to 1000 (**A**).
(EPS)

S8 Fig Model that includes multiple motif hits per region does not affect estimated values for DNA accessibility. A more complex model that includes combined motif score as well as number of motif hits per regions to predict region coverage is tested here. The maximum motif hit score presented in our main model is also part of the more complex model. The correlation between accessibility values predicted in the main model versus the one estimated from the more complex model is above 99.9% (**A**). The more complex model slightly increases correlation between predicted and observed coverage from 0.629 to 0.652. The results are shown by using threshold of minimum coverage per region equal to 10000, which includes 93% of all data points (see S5–S7 Figs).
(EPS)

S9 Fig Our model hypothesizes that *de novo* peak prediction can be estimated in novel experimental conditions without the cost of ChIP-seq experiments for whole TF repertoire. Panels ***A*** and ***B*** illustrate hypothetically the effect of distinct growth conditions on the affinity and accessibility parameters. The TF binding affinity map is sequence specific and does not vary under different experimental conditions (**A**). The accessibility parameter varies among different experimental conditions (**B**). The ChIPseq data used in the main text was collected under the same growth condition. Thus, we assume a unique value for DNA accessibility per region. This may not be the case under other growth conditions. A potential practical application of our model is to use a few set of ChIP-seq experiments to estimate region accessibility and use them in combination with motif score to perform de novo ChIP-seq peak prediction.
(EPS)

S10 Fig The qualitative results from our method is robust to varying value of region bin size. *De novo* peak prediction (top) and correlation of accessibility with distance to *oriC* (bottom) is shown for region bin size of 250bp (**A**) and 1000bp (**B**). The correlation between observed and predicted coverage raises from 0.34 (motif only) to 0.60 (motif + accessibility) and from 0.41 to 0.66 for region bin size of 250 and 1000bp, respectively.
(EPS)

S1 Text Mathematical derivation of gene regulation as a Boltzmann distribution. This supporting text shows a mathematical derivation to describe probability of binding to a genome region as a Boltzmann distribution that depends on two terms: binding accessibility and binding affinity.
(DOCX)
