# Supplementary material for: The Role of Genome Accessibility in Transcription Factor Binding in Bacteria
Source: PLoS Comput Biol. 2016 Apr 22;12(4):e1004891. doi: 10.1371/journal.pcbi.1004891 (PMC4841574; doi:10.1371/journal.pcbi.1004891)
Supplement: S1 Text — This supporting text shows a mathematical derivation to describe probability of binding to a genome region as a Boltzmann distribution that depends on two terms: binding accessibility and binding affinity. (DOCX) [file pcbi.1004891.s011.docx]

**SUPPORTING TEXT S1**

**Mathematical derivation of gene regulation model as a Boltzmann distribution.**

TF-DNA binding can be represented as a collection of DNA regions that may or may not be bound. The DNA lies on a TF reservoir as well as a thermal bath provided by the cytosol. The collection of all possible binding configurations defines the microstates of the system. In statistical mechanics terms, TF-DNA binding is an open system in which DNA regions exchange energy and ligand molecules with the cytoplasm. This situation is formally defined as a Grand Canonical Ensemble [1].

In the following, we show the steps to derive TF-DNA binding in terms of a Boltzmann distribution. Let *p_i,k_* represent the probability a DNA region *i* occurs in binding state *k*. The system is constrained by the average energy, *<E_i_>*, and binding*, <N_i_>* at region *i*. Mathematically, the system is described by the following constraints:

| $\left\{ \begin{aligned} \sum_{k} p_{i,k}=1 \\ \sum_{k} p_{i,k}\varepsilon_{i,k}=<E_{i}> \\ \sum_{k} p_{i,k}N_{i,k}=<N_{i}> \end{aligned} \right.$ | (S1) |
| --- | --- |

where *ε_I,k_* and *N_i,k_* represent the specific energy contribution and the corresponding number of binding molecules at region *i* at state *k*, respectively.

The probabilities *p_i,k_* are obtained by minimizing the free energy of Helmholtz, *F_i_ - μ_i_N_i_= <E_i_> - TdS_i_ -μ_i_ <N_i_*>, where T represents the temperature parameter and $S_{i}=-\kappa\cdot\sum_{k} p_{i,k}\cdot log(p_{i,k})$ is the entropy [1]. The parameter *κ* is the Boltzmann constant. The free energy of Helmholtz is minimized when $d\left( F_{i}-\mu N_{i} \right)=\sum_{k} [\varepsilon_{i,k}+\kappa T(1+\log\left( p_{i,k} \right)\pm\mu_{i}\cdot<N_{i}>]{dp}_{i,k}=0$ constrained to $\sum_{k} p_{i,k}=1$. Using Lagrange multipliers, the probabilities *p_i,k_* is obtained by solving the following equation:

| $\sum_{k} \varepsilon_{i,k}+\kappa T\left( 1+\log\left( p_{i,k} \right)-\mu_{i}<N_{i}>+\lambda\right)dp_{i,k}=0$ | (S2) |
| --- | --- |

Thus:

| $p_{ij}\propto e^{a_{i}+w_{ij}}$ | (S3) |
| --- | --- |

where the affinity parameter *w_i,j_* = *-ε_I,k_ /(κT)* and the accessibility parameter *a_i_* = *μ_i_<N_i_>/(κT).*

1. Dill KA, Bromberg S. Molecular Driving Forces: Statistical Thermodynamics in Chemistry and Biology. 2003:666.
